# Learning efficient navigation in vortical flow fields

**DOI:** 10.1038/s41467-021-27015-y

**Published:** 2021-12-08

**Authors:** Peter Gunnarson, Ioannis Mandralis, Guido Novati, Petros Koumoutsakos, John O. Dabiri

**Affiliations:** 1 Graduate Aerospace Laboratories, California Institute of Technology, 1200 E California Blvd, Pasadena, CA 91125 USA; 2 Computational Science and Engineering Laboratory, ETH Zurich, 8093 Zurich, Switzerland; 3 John A. Paulson School of Engineering and Applied Sciences, Harvard University, 150 Western Ave, Boston, MA 02134 USA; 4 Mechanical and Civil Engineering, California Institute of Technology, 1200 E California Blvd, Pasadena, CA 91125 USA

**Keywords:** Marine biology, Computational science, Mechanical engineering, Biomechanics

## Abstract

Efficient point-to-point navigation in the presence of a background flow field is important for robotic applications such as ocean surveying. In such applications, robots may only have knowledge of their immediate surroundings or be faced with time-varying currents, which limits the use of optimal control techniques. Here, we apply a recently introduced Reinforcement Learning algorithm to discover time-efficient navigation policies to steer a fixed-speed swimmer through unsteady two-dimensional flow fields. The algorithm entails inputting environmental cues into a deep neural network that determines the swimmer’s actions, and deploying Remember and Forget Experience Replay. We find that the resulting swimmers successfully exploit the background flow to reach the target, but that this success depends on the sensed environmental cue. Surprisingly, a velocity sensing approach significantly outperformed a bio-mimetic vorticity sensing approach, and achieved a near 100% success rate in reaching the target locations while approaching the time-efficiency of optimal navigation trajectories.

## Abstract (web summary)

Navigation and trajectory planning in environments with background flow, relevant for robotics, are challenging provided information only on local surrounding. The authors propose a reinforcement learning approach for time-efficient navigation of a swimmer through unsteady two-dimensional flows.

## Introduction

Navigation in the presence of a background unsteady flow field is an important task in a wide range of robotic applications, including ocean surveying^1^, monitoring of deep-sea animal communities^2^, drone-based inspection and delivery in windy conditions^3^, and weather balloon station keeping^4^. In such applications, robots must contend with unsteady fluid flows such as wind gusts or ocean currents in order to survey specific locations and return useful measurements, often autonomously. Ideally, robots would exploit these background currents to propel themselves to their destinations more quickly or with lower energy expenditure.

If the entire background flow field is known in advance, numerous algorithms exist to accomplish optimal path planning, ranging from the classical Zermelo’s equation from optimal control theory^5,6^ to modern optimization approaches^1,3,7–10^. However, measuring the entire flow field is often impractical, as ocean and air currents can be difficult to measure and can change unpredictably. Robots themselves can also significantly alter the surrounding flow field, for example when multi-rotors fly near obstacles^11^ or during fish-like swimming^12^. Additionally, oceanic and flying robots are increasingly operated autonomously and therefore may not have access to real-time external information about incoming currents and gusts (e.g.^13,14^).

Instead, robots may need to rely on data from on-board sensors to react to the surrounding flow field and navigate effectively. A bio-inspired approach is to navigate using local flow information, for example by sensing the local flow velocity or pressure. Zebrafish appear to use their lateral line to sense the local flow velocity and avoid obstacles by recognizing changes in the local vorticity due to boundary layers^15^. Some seal species can orient themselves and hunt in total darkness by detecting currents with their whiskers^16^. Additionally, a numerical study of fish schooling demonstrated how surface pressure gradient and shear stress sensors on a downstream fish can determine the locations of upstream fish, thus enabling energy-efficient schooling behavior^17^.

Reinforcement Learning (RL) offers a promising approach for replicating this feat of navigation from local flow information. In simulated environments, RL has successfully discovered energy-efficient fish swimming^18,19^ and schooling behavior^12^, as well as a time-efficient navigation policy for a repeated, deterministic snapshot of turbulent flow using position information^20^. In application, RL using local wind velocity estimates outperformed existing methods for energy-efficient weather balloon station keeping^4^ and for replicating bird soaring^21^. Other methods exist for navigating uncertainty in a partially known flow field such as fuzzy logic or adaptive control methods^7^. Finite-horizon model predictive control has been also used to plan energy-efficient trajectories using partial knowledge of the surrounding flow field^22^. However, RL can be applied generally to an unknown flow field without requiring human tuning for specific scenarios.

The question remains, however, as to which environmental cues are most useful for navigating through flow fields using RL. A bio-mimetic approach suggests that sensing the vorticity could be beneficial^15^; however flow velocity, pressure, or quantities derived thereof are also viable candidates for sensing.

In this work, we find that Deep RL can indeed discover time-efficient, robust paths through an unsteady, two-dimensional (2D) flow field using only local flow information, where simpler strategies such as swimming towards the target largely fail at the task. We find, however, that the success of the RL approach depends on the type of flow information provided. Surprisingly, a RL swimmer equipped with local velocity measurements dramatically outperforms the bio-mimetic local vorticity approach. These results show that combining RL-based navigation with local flow measurements can be a highly effective method for navigating through unsteady flow, provided the appropriate flow quantities are used as inputs to the algorithm.

## Results

### Simulated navigation problem

As a testing environment for RL-based navigation, we pose the problem of navigating across an unsteady von Kármán vortex street obtained by simulating 2D, incompressible flow past a cylinder at a Reynolds number of 400. Other studies have investigated optimal navigation through real ocean flows^1^, simulated turbulence^20^, and simple flows for which there exist exact optimal navigation solutions^8^. Here, we investigate the flow past a cylinder to retain greater interpretability of learned navigation strategies while remaining a challenging, unsteady navigation problem.

**Fig. 1:**
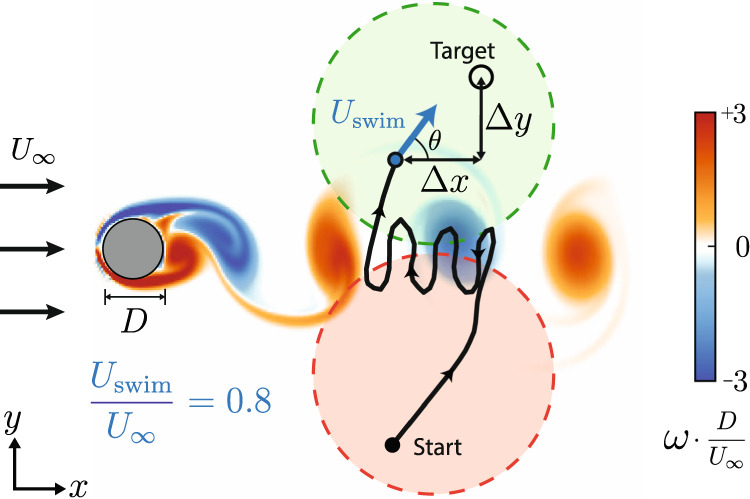
Test navigation problem of navigating through unsteady cylinder flow. Swimmers are initialized randomly inside the red disk and are assigned a random target location inside the green disk. These regions of start and target points are 4*D* in diameter, and are located 5*D* downstream and centered 2.05*D* above and below the cylinder. Additionally, each swimmer is initialized at a random time step in the vortex shedding cycle. An episode is successful when a swimmer reaches within a radius of *D*/6 around the target location.

The swimmer is tasked with navigating from a starting point on one side of the cylinder wake to within a small radius of a target point on the opposite side of the wake region. For each episode, or attempt to swim to the target, a pair of start and target positions are chosen randomly within disk regions as shown in Fig. 1.

Additionally, the swimmer is assigned a random starting time in the vortex shedding cycle. The spatial and temporal randomness prevent the RL algorithm from speciously forming a one-to-one correspondence between the swimmer’s relative position and the background flow, which would not reflect real-world navigation scenarios (see Supplementary Note 1). All swimmers have access to their position relative to the target (Δ*x*, Δ*y*) rather than their absolute position to further prevent the swimmer from relying on memorized locations of flow features during training. For this reason, the start and target regions were chosen to be large relative to the width of the cylinder wake.

For simplicity and training speed, we consider the swimmer to be a massless point with a position **X**_*n*_ = [*x*, *y*] which advects with the time-dependent background flow **U**_flow_ = [*u*(*x*, *y*, *t*), *v*(*x*, *y*, *t*)]. The swimmer can swim with a constant speed ${U}_{{{{{{{{\rm{swim}}}}}}}}}$ and can directly control its swimming direction *θ*. These dynamics are discretized with a time step Δ*t* = 0.3*D*/*U*_*∞*_ using a forward Euler scheme, where *D* is the cylinder diameter and *U*_*∞*_ is the freestream flow velocity:

1 $${{{{{{{{\bf{X}}}}}}}}}_{0}={{{{{{{{\bf{X}}}}}}}}}_{{{{{{{{\rm{start}}}}}}}}},$$

2 $${{{{{{{{\bf{X}}}}}}}}}_{n+1}={{{{{{{{\bf{X}}}}}}}}}_{n}+{{\Delta }}t\left({U}_{{{{{{{{\rm{swim}}}}}}}}}\left[\cos \left(\theta \right),\sin \left(\theta \right)\right]+{{{{{{{{\bf{U}}}}}}}}}_{{{{{{{{\rm{flow}}}}}}}}}\right).$$

It is also possible to apply RL-based navigation with more complex dynamics, including when the swimmer’s actions alter the background flow^12^.

We chose a swimming speed of 80% of the freestream speed *U*_*∞*_ to make the navigation problem challenging, as the swimmer cannot overcome the local flow in some regions of the domain. A slower speed (${U}_{{{{{{{{\rm{swim}}}}}}}}} \, < \, 0.6{U}_{\infty }$) makes navigating this flow largely intractable, while a swimming speed greater than the freestream (${U}_{{{{{{{{\rm{swim}}}}}}}}} \, > \, {U}_{\infty }$) would allow the swimmer to overcome the background flow and easily reach the target.

### Navigation using Deep Reinforcement Learning

In RL, an agent acts according to a policy, which takes in the agent’s state *s* as an input and outputs an action *a*. Through repeated experiences with the surrounding environment, the policy is trained so that the agent’s behavior maximizes a cumulative reward. Here, the agent is a swimmer, the action is the swimming direction *θ*, and we seek to determine how the performance of a learned navigation policy is impacted by the type of flow information contained in the state.

To this end, we first consider a flow-blind swimmer as a baseline, which cannot sense the surrounding flow and only has access to its position relative to the target (*s* = {Δ*x*, Δ*y*}). Next, inspired by the vorticity-based navigation strategy of the zebrafish^15^, we consider a vorticity swimmer with access to the local vorticity at the current and previous time step in order to sense changes in the local vorticity ($s=\left\{{{\Delta }}x,{{\Delta }}y,{\omega }_{n},{\omega }_{n-1}\right.$). We also consider a velocity swimmer, which has access to both components of the local background velocity (*s* = {Δ*x*, Δ*y*, *u*, *v*}). Results for additional swimmers with different states are shown in Supplementary Note 3. In a real robot, velocity sensing could be implemented via a variety of methods including pitot tubes and hot wire or hot film anemometry. Local vorticity could be computed from several velocity sensors. Not considered here are distributed sensing schemes, such as distributed pressure or shear sensors, which can be effective for flow sensing and identification^17^. Coupling optimal flow sensor distribution (e.g.^23^) with the present RL navigation method may be a fruitful, but computationally challenging, extension of this point-swimmer proof of concept.

We employ Deep RL for this navigation problem, in which the navigation policy is expressed using a deep neural network. Previously, Biferale et al.^20^ employed an actor-critic approach for RL-based navigation of a repeated, deterministic snapshot of turbulent flow, which is similar to navigating a steady flow field (see Supplementary Note 1). The policy was expressed using a basis function architecture, requiring a coarse discretization of both the swimmer’s position and swimming direction for computational feasibility. In contrast, V-RACER^24^ is well-suited for this navigation problem, as it is designed for continuous problems and can accept additional sensory inputs with negligible impact in computational complexity. A single 128 × 128 deep neural network is used for the navigation policy, which accepts the swimmers state (i.e. flow information and relative position) and outputs the swimming direction as continuous variables. The network also outputs a Gaussian variance in the swimming direction to allow for exploration during training. The policy network is randomly initialized and then iteratively updated through repeated attempts to reach the target following the policy gradient theorem^25^. V-RACER employs Remember and Forget Experience Replay to reuse past experiences over multiple iterations to update the swimmer’s policy in a stable and data-efficient manner. Additional details of the V-RACER algorithm are shown in Supplementary Note 2. Results such as the success rate and cumulative reward curves were averaged after training each swimmer five times. This step helped ensure that differences in performance did not arise spuriously from the random initialization of the policy network, as described in^26^.

At each time step, the swimmer receives a reward according to the reward function *r*_*n*_, which is designed to produce the desired behavior of navigating to the target. We employ a similar reward function as Biferale et al.^20^:

3 $$\begin{array}{l}{r}_{n}=-{{\Delta }}t+10\left[\frac{| | {{{{{{{{\bf{X}}}}}}}}}_{n-1}-{{{{{{{{\bf{X}}}}}}}}}_{{{{{{{{\rm{target}}}}}}}}}| | }{{U}_{{{{{{{{\rm{swim}}}}}}}}}}-\frac{| | {{{{{{{{\bf{X}}}}}}}}}_{n}-{{{{{{{{\bf{X}}}}}}}}}_{{{{{{{{\rm{target}}}}}}}}}| | }{{U}_{{{{{{{{\rm{swim}}}}}}}}}}\right] +\,{{{{{{{\rm{bonus}}}}}}}}.\end{array}$$

The first term penalizes duration of an episode to encourage fast navigation to the target. The second two terms give a reward when the swimmer is closer to the target than it was in the previous time step. The final term is a bonus equal to 200 time units, or ~30 times the duration of a typical trajectory. The bonus is awarded if the swimmer successfully reaches the target. Swimmers that exit the simulation area or collide with the cylinder are treated as unsuccessful. The second two terms are scaled by 10 to be on the same order of magnitude as the first term, which we found significantly improved training speed and navigation success rates. We also investigated a non-linear reward function, in which the second two terms are the reciprocal of the distance to the target, however it exhibited lower performance. The RL algorithm seeks to maximize the total reward, which is the sum of the reward function across all *N* time steps in an episode:

4 $${r}_{{{{{{{{\rm{total}}}}}}}}}=\mathop{\sum }\limits_{n=1}^{N}{r}_{n}=-{T}_{{{{{{{{\rm{f}}}}}}}}}+10\frac{| | {{{{{{{{\bf{X}}}}}}}}}_{{{{{{{{\rm{start}}}}}}}}}-{{{{{{{{\bf{X}}}}}}}}}_{{{{{{{{\rm{target}}}}}}}}}| | }{{U}_{{{{{{{{\rm{swim}}}}}}}}}}+{{{{{{{\rm{bonus}}}}}}}}.$$

**Fig. 2:**
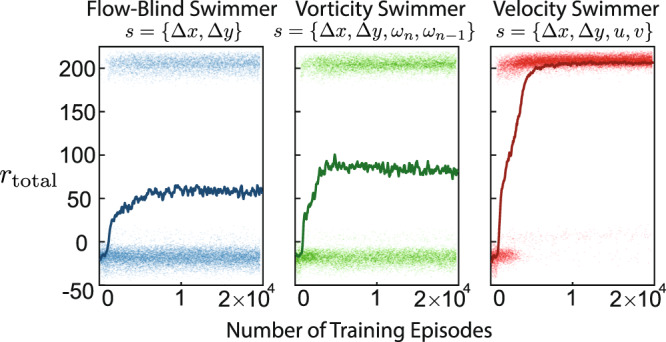
Evolution of the cumulative reward during training for the three RL swimmers. The cumulative rewards for each episode are plotted as points, and a moving average with a window of 201 episodes is plotted with a solid line. Because the swimmer gains a bonus of 200 for reaching the target, successful episodes are clustered around a reward of 200 while unsuccessful episodes are clustered below zero.

The evolution of *r*_total_ during training for each swimmer is shown in Fig. 2. All RL swimmers were trained for 20,000 episodes.

The reward function can be tuned to optimize for specific objectives such as minimum fuel consumption by including additional terms (e.g.^27^). Here, the reward function acts to optimize for two objectives: minimal arrival time to the target (−*T*_f_) and maximum success rate of reaching the target (second two terms). The ability of RL to achieve these two objectives is explored in the following sections.

### Success of RL navigation

**Fig. 3:**
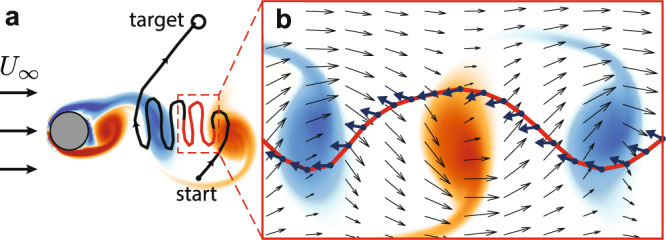
Example trajectory of the velocity RL swimmer. **a** Trajectory plotted in a cylinder-fixed frame, showing the swimmer successfully navigate from its starting location to the target. **b** Segment of this trajectory plotted in a wake-stationary frame of reference on top of the background flow field, which highlights the swimmer exploiting low-velocity regions in the cylinder wake to swim upstream. The swimming direction is plotted at each time step along the trajectory, revealing that this RL swimmer adjusts it swimming direction in response to the changing background flow, enabling time-efficient navigation.

After training, Deep RL discovered effective policies for navigating through this unsteady flow. An example of a path discovered by the velocity RL swimmer is shown in Fig. 3. Because the swimming speed is less than the freestream velocity, the swimmer must utilize the wake region where it can exploit slower background flow to swim upstream. Once sufficiently far upstream, the swimmer can then steer towards the target. The plot of the swimming direction inside the wake (Fig. 3b) shows how the swimmer changes its swimming direction in response to the background flow, enabling it to maintain its position inside the wake region and target low-velocity regions.

**Fig. 4:**
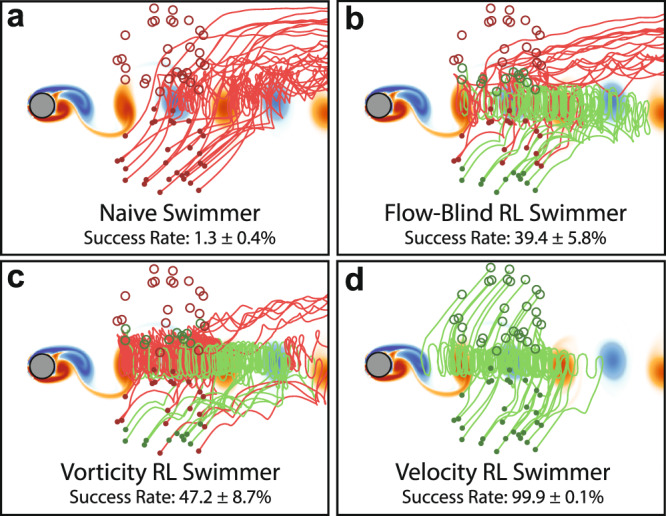
Average success rate with 30 example trajectories for each swimmer type. Successful attempts to reach the target are green, while unsuccessful attempts are red. **a** Naive policy of swimming towards the target is rarely successful. **b** The flow-blind RL swimmer navigates more effectively than the naive swimmer. **c** The vorticity RL swimmer is more successful than the flow-blind swimmer, showing that sensing the local flow can improve RL-based navigation. **d** Surprisingly, the velocity RL swimmer nearly always reaches the target using only the local flow velocity. The stated success rates are averaged over 12,500 episodes and are shown with one standard deviation arising from the five times each swimmer was trained.

However, the ability of Deep RL to discover these effective navigation strategies depends on the type of local flow information included in the swimmer state. To illustrate this point, example trajectories and the average success rates of the flow-blind, vorticity, and velocity RL swimmers are plotted in Fig. 4, and are compared with a naive policy of simply swimming towards the target (*θ*_naive_ = ${\tan }^{-1}\left({{\Delta }}y/{{\Delta }}x\right)$). An example trajectory from each swimmer is also shown in Supplementary Video 1.

A naive policy of swimming towards the target is highly ineffective. Swimmers employing this policy are swept away by the background flow, and reached the target only 1.3% of the time on average. A RL approach, even without access to flow information, is much more successful: the flow-blind swimmer reached the target locations nearly 40% of the time.

Giving the RL swimmers access to local flow information increases the success further: the vorticity RL swimmer averaged a 47.2% success rate. Surprisingly however, the velocity swimmer has a near 100% success rate, greatly outperforming the zebrafish-inspired vorticity approach. With the right local flow information, it appears that an RL approach can navigate nearly without fail through a complex, unsteady flow field. However, the question remains as to why some flow properties are more informative than others.

**Fig. 5:**
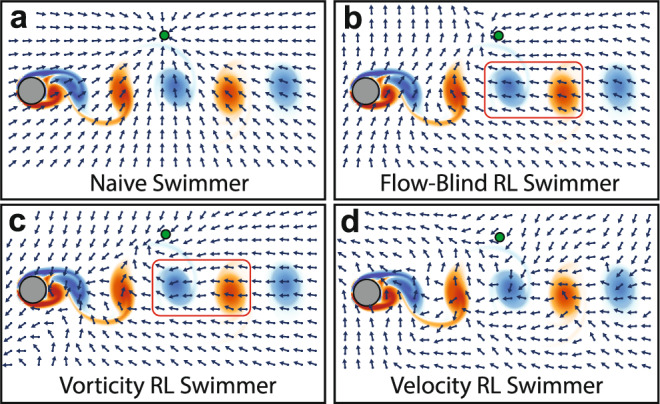
Swimming direction policy plotted across the domain for a fixed target (green circle) at a given time instant. **a** The naive swimmer swims towards the target. **b** The red outline highlights how the flow-blind swimmer navigates irrespective of the background flow, while the vorticity swimmer **c** adjusts its swimming direction modestly. **d** The velocity swimmer appears even more sensitive to the unsteady background flow.

To better understand the difference between RL swimmers with access to different flow properties, the swimming direction computed by each RL policy is plotted over a grid of locations in Fig. 5. The flow-blind swimmer does not react to changes in the background flow field, although it does appear to learn the effect of the mean background flow, possibly through correlation between the mean flow and the relative position of the swimmer in the domain. This provides it an advantage over the naive swimmer. The vorticity swimmer adjusts its swimming direction modestly in response to changes in the background flow, for example by swimming slightly upwards in counter-clockwise vortices and slightly downwards in clockwise vortices. The velocity swimmer appears most sensitive to the background flow, which may help it respond more effectively to changes in the background flow.

Station-keeping inside the wake region may be important for navigating through this flow. In the upper right of the domain, the velocity swimmer learns to orient downwards and back to the wake region, while the other swimmers swim futilely towards the target. Because the vorticity depends on gradients in the background flow, that property cannot be used to respond to flow fields that are spatially uniform. These differences appear to explain many of the failed trajectories in Fig. 4, in which the flow-blind and vorticity swimmers are swept up and to the right by the background flow. Other swimmers with partial access to the background flow fared similarly to the vorticity swimmer, further suggesting that sensing both velocity components are required for best performance (see Supplementary Note 3).

While sensing of point vorticity is insufficient to detect spatially uniform flow fields, it can be useful for distinguishing the vortical wake from the freestream flow. This can explain why the vorticity swimmer performs better than the flow-blind swimmer. A similar reasoning could apply to swimmers that sense other flow quantities such as pressure or shear. Indeed, Alsalman et al. found that velocity sensors outperformed vorticity sensors for neural network-based flow classification^28^.

In addition to providing environmental cues, however, the background flow velocity may be particularly important for navigation, as it affects the future state of the swimmer. Because the flow advects the swimmers according to linear dynamics (Equation (2)), the local velocity can exactly determine the swimmer’s position at the next time step. This may explain the high navigation success of the velocity swimmer, as it has the potential to accurately predict its next location. To be sure, the Deep RL algorithm must still learn where the most advantageous next location ought to be, as the flow velocity at the next time step is still unknown.

For real swimmers, vorticity may also affect the future state of the swimmer, for example by causing a swimmer to rotate in the flow^29^ or by altering boundary layers and skin friction drag^12^. Real robots would also be subject to additional sources of complexity not considered in this simplified simulation, which would make it more difficult to determine a swimmer’s next position from local velocity measurements alone.

### Comparison with optimal control

In addition to reaching the destination successfully, it is desirable to navigate to the target while minimizing energy consumption or time spent traveling. Biferale et al.^20^ demonstrated that RL can approach the performance of time-optimal trajectories in steady flow for fixed start and target positions. Here, we find that this result also holds for the more challenging problem of navigating unsteady flow with variable start and target points.

Assuming the swimmer reaches the target location, the only term in the cumulative reward *r*_total_ that depends on the swimmer’s trajectory is −*T*_f_ (Equation (4)). Therefore, maximizing the cumulative reward of a successful episode is equivalent to finding the minimum time path to the target. Because the velocity RL swimmer always reaches the target successfully, we compare the velocity RL swimmer to the time-optimal swimmer derived from optimal control.

To find time-optimal paths through the flow, given knowledge of the full velocity field at all times, we constructed a path planner that finds locally optimal paths in two steps. First, a rapidly-exploring random tree algorithm (RRT) finds a set of control inputs that drive the swimmer from the starting location to the target location, typically non-optimally^30^. Then we apply constrained gradient-descent optimization (i.e. the fmincon function in MATLAB) to minimize the time step (and therefore overall time *T*_f_) of the trajectory while enforcing that the swimmer starts at the starting point (Equation (1)), obeys the dynamics at every time step in the trajectory (Equation (2)), and reaches the target (∣∣**X**_*N*_ − **X**_target_∣∣ < = *D*/6). The trajectories produced by this method are local minima, so the fastest trajectory was chosen out of 100 runs and validated to be globally optimal by comparing it with the output of the level set method described in Lolla et al.^10^ computed using a MATLAB level set toolbox^31^. Other algorithms could also be used to find optimal trajectories for unsteady flow given knowledge of the entire flow field^8^.

**Fig. 6:**
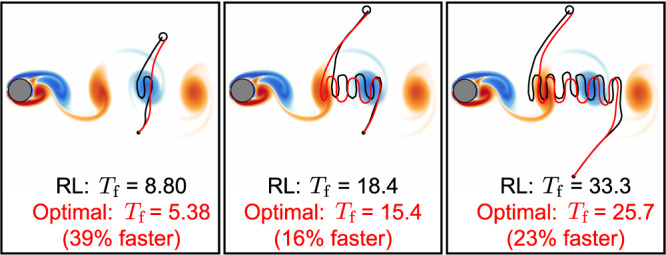
Comparison between time-optimal and RL trajectories. Time-optimal trajectories are shown in red and RL trajectories are shown in black. The RL swimmer used the state *s* = {Δ*x*, Δ*y*, *u*, *v*}. Time to reach the target *T*_f_ is made non-dimensional using the timescale *D*/*U*_*∞*_.

A comparison between RL and time-optimal navigation for three sets of start and target points is shown in Fig. 6. These points were chosen to represent a range of short and long duration trajectories. Despite only having access to local information, the RL trajectories are nearly as fast and qualitatively similar to the optimal trajectories, which were generated with the advantage of having full global knowledge of the flow field. A comparison between the swimmers is also shown in Supplementary Video 2.

The surprisingly high performance of the RL approach compared to a global path planner suggests that deep neural networks can, to some extent, approximate how local flow at a particular time impacts navigation in the future. In other words, a successful RL swimmer must simultaneously navigate and identify the approximate current state of the environment using only a single flow measurement at one instant in time at an unknown absolute location in the flow field. In comparison, the optimal control approach relies on knowledge of the environment in advance. There are limitations to the RL approach, however. For example, the optimal swimmer on the right of Fig. 6 enters the wake region at a different location than the RL swimmer to avoid a high velocity region, which the RL swimmer may not have been able to sense initially.

In addition to approaching the optimality of a global planner, RL navigation offers a robustness advantage. As noted in^20^, RL can be robust to small changes in initial conditions. Here, we show that RL navigation can generalize to a large area of initial and target conditions as well as random starting times in the unsteady flow. Additionally, we found that the velocity RL swimmer is robust to realistic amounts of sensor noise from turbulent fluctuations (see Supplementary Note 4).

In contrast, the optimal trajectories here are open loop: any disturbance or flow measurement inaccuracy would prevent the swimmer from successfully navigating the target. While robustness can be included with optimal control in other ways^7^, responding to changes in the surrounding environment is the driving principle of this RL navigation policy. Indeed, the related algorithm of imitation learning has been used for drone control by employing a neural network to mimic an optimal flight path while reacting to local disturbances^32^.

### Policy transfer to double gyre flow

The RL swimmer showed robustness to large changes in the start and target positions, and to realistic amounts of sensor noise (Supplementary Note 4). However, it is worth considering if a learned navigation policy can transfer between different flow fields, which would reduce the amount of training required for navigating a new flow field and increase the robustness of a swimmer to sudden changes in its environment.

Colabrese et al. demonstrated that an RL swimmer trained on a vortical flow field can navigate successfully in a new, but topologically similar, flow field without additional training^29^. However, they noted that learned navigation strategies may not transfer between dissimilar flows, thus requiring additional training to form a new navigation strategy. Here, we consider if the learned policy for navigating the cylinder flow can transfer to a double gyre flow, which is topologically dissimilar.

**Fig. 7:**
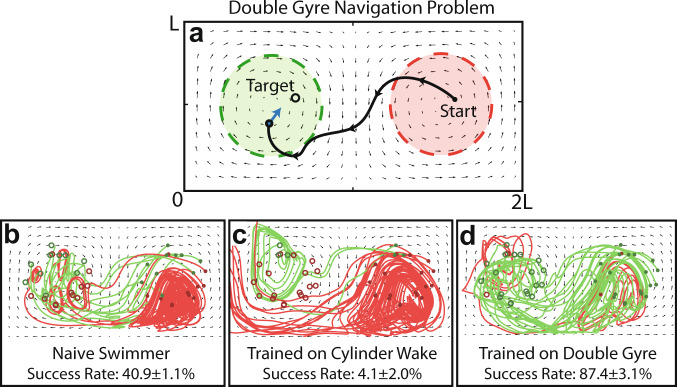
RL navigation in the double gyre flow field. **a** Navigation problem setup. The start and target regions are *L*/2 in diameter and located at (3*L*/2, *L*/2) and (*L*/2, *L*/2), respectively. **b** A naive policy achieves 40.9% success rate on average. **c** The velocity RL swimmer trained on the cylinder wake navigates the double gyre flow poorly, indicating its navigation policy did not generalize. **d** After receiving training for the double gyre flow, the velocity RL swimmer is able to adapt and navigate more effectively than either swimmer. As with the cylinder flow, successful attempts to reach the target are green, while unsuccessful attempts are red. An episode is successful when a swimmer reaches within a radius of *L*/50 around the target location. The stated success rates are averaged over 12,500 episodes and are shown with one standard deviation arising from the five times each swimmer was trained.

The double gyre flow is a 2D, unsteady, periodic flow field that is a simplified representation of circulation patterns found frequently in the ocean^22,33,34^. The velocity field is defined analytically in^33^, where all length units are non-dimensional (i.e. *L* = 1). Here, we used $A=2/3{U}_{{{{{{{{\rm{swim}}}}}}}}}$, *ϵ* = 0.3, and $\omega =20\pi {U}_{{{{{{{{\rm{swim}}}}}}}}}/3L$, which presents a challenging navigation problem that is unsteady on a similar time scale as the cylinder flow. Swimmers were started at a random time step in the right gyre and are tasked with navigating to a randomly chosen target in the left gyre. The problem setup is shown in Fig. 7a.

To see if the learned RL policy transfers to the double gyre flow, two versions of the velocity RL swimmer were tested: one trained on the unsteady cylinder flow and one trained for the double gyre flow. Additionally, the naive swimmer was included for comparison. The success rates of these swimmers are shown in Fig. 7b–d.

The learned policy for navigating the cylinder wake did not transfer effectively to the double gyre flow, resulting in only a 4.1% average success rate (Fig. 7c) compared to the naive swimmer’s 40.9% average success rate (Fig. 7b). Poor performance was also observed when the problem coordinates were rotated and scaled to match the start and target regions of the cylinder flow navigation problem.

With training, however, new and effective navigation strategies can be learned. The velocity RL swimmer trained on the double gyre flow achieved a high average success rate of 87.4%, leveraging the background flow to escape the right gyre and navigate to its target locations in the left gyre. These results suggest that learned policies may indeed only transfer between similar flows, and that effective navigation in new flow fields requires additional training. Additionally, while all investigations here are in simulated flow environments, future studies may benefit from investigating the transfer of learned behaviors between simulated and real environments, which can reduce in situ training time for physical robots.

## Discussion

We have shown in this study how Deep RL can discover robust and time-efficient navigation policies which are improved by sensing local flow information. A bio-inspired approach of sensing the local vorticity provided a modest increase in navigation success over a position-only approach, but surprisingly the key to success was discovered to lie in sensing the velocity field, which more directly determined the future position of the swimmer. This suggests that RL coupled with an on-board velocity sensor may be an effective tool for robot navigation. While the learned policy for navigating an unsteady cylinder wake did not transfer to a dissimilar double gyre flow, additional training enabled the RL swimmer adapt to the new flow field. Future investigation is warranted to examine the extent to which the success of the velocity approach extends to real-world scenarios, in which robots may face more complex, 3D fluid flows, and be subject to non-linear dynamics and sensor errors.

## Supplementary information

Supplementary Information

Description of additional Supplementary File

Supplementary Video 1

Supplementary Video 2

## Data Availability

All data generated and discussed in this study are available within the article and its supplementary files, or are available from the authors upon request.

## Code availability

The Deep Reinforcement Learning algorithm V-RACER is available at github.com/cselab/smarties.
