# Supplementary material for: Learning efficient navigation in vortical flow fields
Source: Nat Commun. 2021 Dec 8;12:7143. doi: 10.1038/s41467-021-27015-y (PMC8654940; doi:10.1038/s41467-021-27015-y)
Supplement: Supplementary file 1 — Supplementary Information [file 41467_2021_27015_MOESM1_ESM.pdf]

Learning Efficient Navigation in Vortical Flow Fields
Supplementary Notes

SUPPLEMENTARY NOTE 1 - NAVIGATION IN STEADY FLOW

For navigating through an unsteady von Kármán vortex street, flow sensing appeared critical for learning effective swimming strategies with Reinforcement Learning. For navigating steady flows with a fixed target, however, an RL-swimmer may be able to navigate simply by forming a one-to-one correspondence between its position and the fixed background flow field.

Consider the steady 2D flow past a cylinder at a Reynolds number of 40. Here, the target position is fixed to be just downstream of the cylinder, and the swimmers are started randomly throughout the entire domain with a swimming speed of $U_{\text{swim}} = 0.5U_\infty$ (see Supplementary Figure 2). Successful swimmers generally start close enough to the cylinder to use the wake to navigate to the target, while unsuccessful swimmers generally start too far away from the cylinder to reach the target before being swept downstream.

We trained a flow-blind swimmer ($s = \{\Delta x, \Delta y\}$) and a vorticity swimmer ($s = \{\Delta x, \Delta y, \omega_n, \omega_{n-1}\}$). Even though the flow-blind swimmer had no knowledge of the background flow, it trained as quickly as the vorticity swimmer, which can be seen in the evolution of the cumulative reward over training (Supplementary Figure 1).

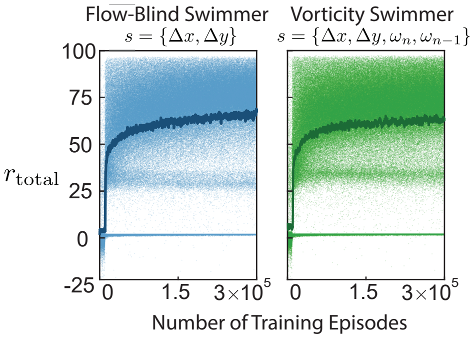

Supplementary Figure 1. Evolution of the cumulative reward for a flow-blind swimmer and a vorticity swimmer in steady flow past a cylinder. Because the background flow is steady and the target location is fixed, both the flow-blind and vorticity swimmers train at a similar rate.

Additionally, both swimmers were equally successful at reaching the target after training, which is visualized by plotting the region of starting points from which the swimmer can reach the target (Supplementary Figure 2). This region is computed by extracting the ridges of the finite-time lyapunov exponent field [1] of the flow field formed by the background flow plus the swimmer's learned policy.

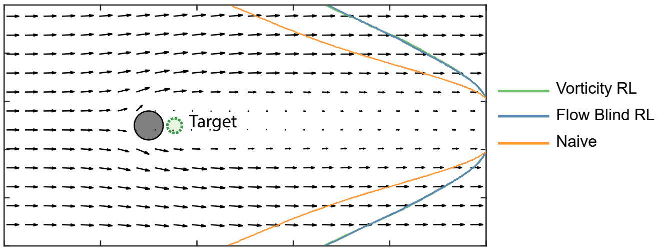

Supplementary Figure 2. Success of a naïve swimmer, flow-blind RL swimmer, and vorticity RL swimmer at navigating the steady flow past a cylinder. The plotted curves display the right-hand (i.e. downstream) boundary of the region in which the swimmer can reach the target. This region of attraction to the target is identical for both RL swimmers, and larger than the region for the naïve swimmer. This implies that in this steady flow field, both RL swimmers are equally successful at reaching the target despite one swimmer lacking flow sensing abilities.

Because an RL swimmer can navigate steady flow with position alone, the unsteady cylinder wake was chosen for testing flow sensing-based navigation. Additionally, the starting time was randomized, as presenting a swimmer with a repeated, deterministic snapshot of a flow field could also be navigable by memorizing the flow based on position alone.

SUPPLEMENTARY NOTE 2 - REINFORCEMENT LEARNING ALGORITHM

We employed the V-RACER algorithm for training the deep RL swimmers using the smarties framework. Some details of the algorithm are presented here, but a complete description can be found in [2].

The goal of V-RACER is to train the weights w of a neural network using experiences with the environment. At each time-step t, the neural network takes in the agent's state s_t as an input and outputs the mean action μ^{w}, standard deviation σ^{w}, and value estimate v^{w}. During training, the swimmer takes an action a_t sampled from the normal distribution $\mathcal{N}(\mu^{\text{w}}, (\sigma^{\text{w}})^2)$. The architecture of this deep neural network is shown below in Supplementary Figure 3.

At every time step in an episode, the agent takes the action a_t and stores information such as the state (s_t), reward information ($r_t, \hat{V}_t^{\text{tbc}}$), the current policy ($\mu_t, \sigma_t$), and action it took ($a_t$) in the Replay Memory (RM). The number of recorded experiences is kept to a fixed size: as new experiences are added, the oldest ones are forgotten.

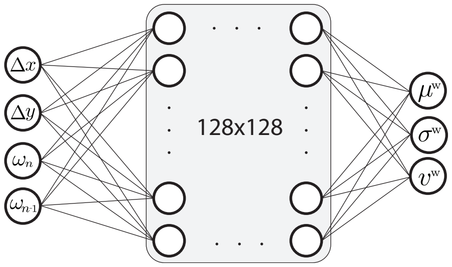

Supplementary Figure 3. Policy network used for deep RL with V-RACER. The state (e.g. $s = \{\Delta x, \Delta y, \omega_n, \omega_{n-1}\}$) is inputted into a deep neural network and the output is a mean action μ^{w}, standard deviation σ^{w}, and value v^{w}. During training, the swimmer chooses a swimming angle by random sampling from a normal distribution: $\theta \sim \mathcal{N}(\mu^{\mathrm{w}}, (\sigma^{\mathrm{w}})^2)$. After training, the mean action is selected ($\theta = \mu^{\mathrm{w}}$).

To update the network's weights and biases, experiences are sampled from the RM, and a gradient is computed for each past experience according to two loss functions. The value output v of the neural network is trained using the following loss function, which seeks to improve the neural network's value estimate $v^{\mathrm{w}}(s_t)$ to better match the estimated value from experiences $\hat{V}_t^{tbc}$:

$$L^{\mathrm{ret}}(w) = \frac{1}{2}\left(v^{\mathrm{w}}(s_t) - \hat{V}_t^{tbc}\right)^2. \quad (1)$$

The mean and standard deviation outputs (μ^{w} and σ^{w}) are trained using the following loss function, which seeks to change the policy to improve on-policy returns:

$$L^{\mathrm{off-PG}}(\mathrm{w}) = -\rho_t^{\mathrm{w}}\left(r_{t+1} + \gamma \hat{V}_{t+1}^{tbc} - v^{\mathrm{w}}(s_t)\right), \quad (2)$$

where the importance weight ρ_t^{w} is the ratio of the probability of selecting a_t given s_t using the current policy $\pi(\cdot \mid s_t) \sim \mathcal{N}(\mu^{\mathrm{w}}, (\sigma_t^{\mathrm{w}})^2)$ and the old policy $\beta(\cdot \mid s_t) \sim \mathcal{N}(\mu_t, \sigma_t^2)$:

$$\rho_t^{\mathrm{w}} = \frac{\pi^{\mathrm{w}}(a_t \mid s_t)}{\beta(a_t \mid s_t)}. \quad (3)$$

The estimate of the return $\hat{V}_t^{tbc}$ is calculated using a recursive formula starting at the terminal time step N of an episode in the RM and stepping backwards:

$$\hat{V}_N^{tbc} = 0, \quad (4)$$

$$\hat{V}_t^{tbc} = v^{\mathrm{w}}(s_t) + \min\{1, \rho_t^{\mathrm{w}}\}\left[r_{t+1} + \gamma \hat{V}_{t+1}^{tbc} - v^{\mathrm{w}}(s_t)\right]. \quad (5)$$

The gradient estimate is then:

$$\hat{g}_{t_i} = \frac{\partial L^{\mathrm{ret}}}{\partial v^{\mathrm{w}}}\nabla v^{\mathrm{w}} + \frac{\partial L^{\mathrm{off-PG}}}{\partial \mu^{\mathrm{w}}}\nabla \mu^{\mathrm{w}} + \frac{\partial L^{\mathrm{off-PG}}}{\partial \sigma^{\mathrm{w}}}\nabla \sigma^{\mathrm{w}}. \quad (6)$$

If the old policy is too dissimilar from the current policy ($1/c_{max} < \rho_{t_i}^{\mathrm{w}} < c_{max}$), $\hat{g}_{t_i}$ is set to zero. Additionally, this gradient estimate is mixed with a gradient that points in the direction of the current policy to prevent the policy from changing too greatly in an unstable manner:

$$\hat{g}_{t_i}^{ReF-ER} = \lambda \hat{g}_{t_i} + (1-\lambda)\nabla D_{\mathrm{KL}}(\beta(\cdot \mid s_t) \mid\mid \pi(\cdot \mid, s_t)), \quad (7)$$

where D_{KL} is the Kullback–Leibler divergence, and λ is a chosen parameter. For the RL swimmers, the neural network was updated using $\hat{g}_{t_i}^{ReF-ER}$ at each time step. Additional details, such as psuedocode, hyperparameters, and the scaling of the neural network inputs are shown in [2].

A network size of 128×128 was selected to ensure the network would be sufficiently expressive to learn effective navigation strategies. To confirm that a 128×128 is sufficiently expressive, we found that a network size of 64×64 was able to match the performance of a 128×128 network, which can be see below in Supplementary Figure 4.

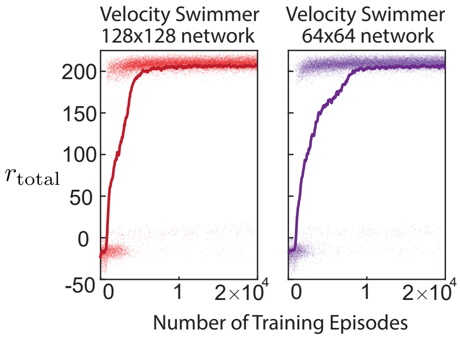

Supplementary Figure 4. Evolution of the cumulative reward for a velocity swimmer with a 128×128 neural network and a 64×64 neural network, which shows that a 128×128 neural network is sufficiently large to express an effective swimming policy.

SUPPLEMENTARY NOTE 3 - ADDITIONAL RL SWIMMERS

In addition to flow-blind, vorticity, and velocity RL swimmers, several other swimmers with different states were investigated. The u-velocity swimmer has access to the only the x component of the flow velocity ($s = \{\Delta x, \Delta y, u\}$). The transverse-velocity swimmer has access to the component of the fluid velocity perpendicular to the swimmer's previous direction of travel ($s = \{\Delta x, \Delta y, u_\perp, v_\perp\}$). Finally, the vorticity-velocity swimmer has access to the both the vorticity and velocity of the fluid ($s = \{\Delta x, \Delta y, \omega_n, \omega_{n-1}, u, v\}$).

The success rate of all swimmers is plotted in Supplementary Figure 5. All swimmers with access to partial-flow information (e.g. vorticity, one velocity component)

have a slightly higher success rate than the flow-blind swimmer. The two swimmers with access to both components of the fluid velocity reach a nearly 100 percent success rate, and the inclusion of vorticity in addition to the flow velocity did not appear to impact training time.

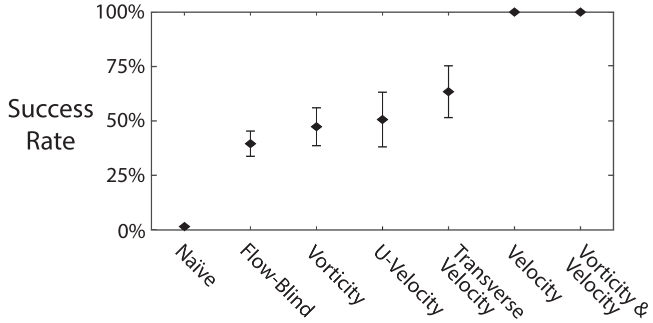

Supplementary Figure 5. Success rates of all investigated swimmers with various local sensing schemes. The stated success rates are averaged over 12,500 episodes and are shown with one standard deviation arising from the five times each swimmer was trained.

SUPPLEMENTARY NOTE 4 - SENSOR NOISE

All swimmers have thus far been presented noiseless measurements of the background flow and the swimmer's position. In a biological context, such low-noise measurements may not be unreasonable: seals were reported to detect flow velocities as low as 245 microns per second using specially adapted whiskers [3]. In robotic systems, however, measurement noise could arise from a variety of sources, such as a measurement device itself, or from small scale turbulence when navigating with background flow. Given that turbulence is ubiquitous in real-world flows and the RL swimmers in the present study rely on background flow measurements to navigate, we investigated how noise in the flow measurements affects the success rate of RL swimmers. We found that the velocity swimmer can be robust to realistic amounts of flow sensing noise.

To simulate flow measurement noise, an already trained velocity RL swimmer was tasked with navigating across the unsteady cylinder wake with zero-mean Gaussian noise added to both components of its velocity measurement. The standard deviation of the velocity sensor noise, σ_{sensor}, was varied between 0 and 0.5 times U_∞. The success rates of the velocity swimmer for various amounts of noise are shown in Supplementary Figure 6.

The velocity RL swimmer demonstrated robustness to noise in its local velocity measurement, showing little decrease in the success rate with a σ_{sensor} of up to 10 percent of the freestream flow velocity. With higher amounts of noise, the success rate decreased, although the velocity

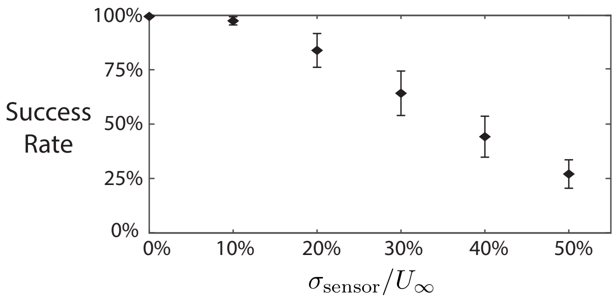

Supplementary Figure 6. Success rates of a velocity RL swimmer with various amounts of zero-mean Gaussian noise added to its local velocity measurement (position measurements were left noiseless). The stated success rates are averaged over 12,500 episodes and are shown with one standard deviation arising from the five times each swimmer was trained.

swimmer can still navigate more successfully than flow-blind swimmer even when σ_{sensor} reaches 40 percent of U_∞. It is not surprising that velocity measurements are less useful at this noise level, because the noise is comparable to the measured signal.

A convenient, albeit crude, metric for comparison with real fluid flows is the turbulence intensity, defined as the ratio of the root-mean squared velocity fluctuations over the mean velocity ($u_{\text{rms}}/\bar{U}$). While turbulence does not exactly follow a Gaussian distribution [4] and not all scales of turbulence would be small enough to appear as random fluctuations to a swimmer [5], we can compare the turbulence intensity of real flows to the random noise added to the swimmer's sensor readings.

Typical turbulence intensities used for the design of underwater autonomous vehicles can range from 0.2 to 9 percent [6, 7]. For tidal flows in the ocean, the turbulent intensity has been measured to be approximately 12 to 13 percent [8] but could be higher near surface waves or lower in the mid-ocean. For aerial vehicles, typical turbulence intensities could range from 1.2 to 12.6 percent [5]. By comparison, the RL swimmer has a minimal decrease in its success rate with a similar magnitude of random noise in its velocity measurement. To be sure, the turbulence intensity can range from 0 to infinity depending on the presence of a mean background current, and can vary in magnitude for different velocity components [5], so this comparison is only a rough approximation of in situ noise levels. A physical implementation of RL navigation with real-world noise would provide a more conclusive result, but it is nevertheless promising that perfectly noiseless flow measurements are not required for high navigation success in the simulated case.

SUPPLEMENTARY REFERENCES

[1] S. C. Shadden, F. Lekien, and J. E. Marsden, Physica D: Nonlinear Phenomena **212**, 271 (2005).

[2] G. Novati and P. Koumoutsakos, arXiv:1807.05827 [cs, stat] (2019), arXiv: 1807.05827.

[3] G. Dehnhardt, B. Mauck, and H. Bleckmann, Nature **394**, 235 (1998), number: 6690 Publisher: Nature Publishing Group.

[4] J. Jiménez (2006).

[5] S. Watkins, S. Ravi, and B. Loxton, Engineering Letters **18**, 6 (2009), place: Hong Kong Publisher: International Association of Engineers.

[6] A. K. Lidtke, S. R. Turnock, and J. Downes, in *2016 IEEE/OES Autonomous Underwater Vehicles (AUV)* (IEEE, Tokyo, Japan, 2016) pp. 364–373.

[7] W. Tian, Z. Mao, F. Zhao, and Z. Zhao, Complexity **2017**, 1 (2017).

[8] I. A. Milne, R. N. Sharma, R. G. J. Flay, and S. Bickerton, Philosophical Transactions of the Royal Society A: Mathematical, Physical and Engineering Sciences **371**, 20120196 (2013), publisher: Royal Society.
