# Supplementary material for: Learning efficient navigation in vortical flow fields
Source: Nat Commun. 2021 Dec 8;12:7143. doi: 10.1038/s41467-021-27015-y (PMC8654940; doi:10.1038/s41467-021-27015-y)
Supplement: Supplementary file 2 — Description of additional Supplementary File [file 41467_2021_27015_MOESM2_ESM.pdf]

Description of additional supplementary data files

Supplementary Video 1: Example trajectories for each swimmer type.

Supplementary Video 2: Comparison between optimal and RL swimmer.
